# Early initiated noradrenaline versus fluid therapy for hypotension and shock in the emergency department (VASOSHOCK): a protocol for a pragmatic, multi-center, superiority, randomized controlled trial

**DOI:** 10.1186/s13049-025-01369-4

**Published:** 2025-04-07

**Authors:** Lasse Paludan Bentsen, Thomas Strøm, Jakob Lundager Forberg, Gerhard Tiwald, Peter Biesenbach, Malik Kalmriz, Jens Henning Rasmussen, Nikolaj Raaber, Sören Möller, Mette Løkke, Gitte Boier Tygesen, Hanne Nygaard, Josephine Hyldgaard Brok, Julie Westergaard Andersen, Nikolett Bajusz, Mikkel Brabrand

**Affiliations:** 1 Department of Emergency Medicine, Odense University Hospital, Odense, Denmark; 2 Research Unit for Emergency Medicine, Odense University Hospital, Odense, Denmark; 3 Department of Clinical Research, University of Southern Denmark, Odense, Denmark; 4 Department of Emergency Medicine, Lillebaelt Hospital, Kolding, Denmark; 5 Department of Anaesthesia and Intensive Care Medicine, Odense University Hospital, Odense, Denmark; 6 Department of Anaesthesia and Intensive Care Medicine, Hospital of Southern Jutland, Aabenraa, Denmark; 7 Department of Emergency Medicine, Helsingborg Hospital, Helsingborg, Sweden; 8 Department of Clinical Sciences, Lund University, Lund, Sweden; 9 Emergency Department, Zealand University Hospital, Køge, Denmark; 10 Department of Emergency Medicine, Esbjerg University Hospital, Esbjerg, Denmark; 11 Research Unit of Emergency Medicine, Esbjerg University Hospital, Esbjerg, Denmark; 12 Emergency Department, Gødstrup Regional Hospital, Herning, Denmark; 13 Department of Emergency Medicine, Copenhagen University Hospital, Bispebjerg and Frederiksberg Hospital, Copenhagen, Denmark; 14 Department of Emergency Medicine, Aarhus University Hospital, Aarhus, Denmark; 15 Open Patient Exploratory Network (OPEN), University of Southern Denmark and Odense University Hospital, Odense, Denmark; 16 Research Center for Emergency Medicine, Department of Clinical Medicine, Aarhus University, Aarhus, Denmark

**Keywords:** Shock, Hypotension, Vasopressor, Norepinephrine, Noradrenaline, Fluid therapy, Resuscitation, Emergency medicine, Emergency department

## Abstract

**Background:**

Shock is a condition with high mortality even with early intervention and treatment. Usual care for shock and hypotension in the Emergency Department (ED) is intravenous fluid resuscitation which can lead to fluid overload and other complications. When fluid therapy fails or risk of complications are high, the next treatment step is the use of vasopressors for stabilisation. Noradrenaline therapy for hypotension and shock are commonly used in ED’s outside Scandinavia, but the evidence on the optimal initiation time is sparse. The lack of noradrenaline therapy in Scandinavia provides a unique environment to investigate the possible implications of early initiation. The aim of this trial is to investigate whether the use of early initiated noradrenaline compared to ED fluid therapy can improve blood pressure goals and by that, reduce the need for ICU admittance.

**Methods:**

This protocol describes a pragmatic, multi-center, superiority randomized controlled trial, randomizing patients with hypotension to intervention or control. Eligible patients are ≥ 18-year-old who have received at least 500 ml intravenous fluids (including prehospital administration), and without suspected cardiogenic, haemorrhagic, anaphylactic, or neurogenic causes, or require direct ICU admittance due to non-hemodynamic severe organ failure. The intervention group receives noradrenaline initiated at 0.05 mcg/kg/min with a maximum of 0.15 mcg/kg/min through a peripheral venous catheter for up to 24 h. The control group receives usual care. Treatment is targeted for a systolic blood pressure ≥ 100 mmHg, a mean arterial pressure ≥ 65 mmHg or a clinician defined blood pressure target. We require a sample size of 320 patients to show a significant difference in proportion of patients achieving shock control within 90 min (primary endpoint). Key secondary outcomes include ICU free days alive within 30-days and 30-day all-cause mortality.

**Discussion:**

Previous prospective randomized trials on early peripheral noradrenaline treatment for shock are sparse and are investigated in settings where noradrenaline use is already usual care. Since noradrenaline are not used as standard treatment for shock in Scandinavian EDs, this provides a unique opportunity not only to investigate the early initiation of noradrenaline for shock, but also comparing it directly to ED fluid only approach.

*Trial registration*: EU CT ID 2023-504584-16-00. ClinicalTrials.gov NCT05931601. URL: https://classic.clinicaltrials.gov/ct2/show/NCT05931601

**Supplementary Information:**

The online version contains supplementary material available at 10.1186/s13049-025-01369-4.

## Introduction

Hypotension and shock are relatively common conditions, present in about 1.2% of patients seen in the Emergency Department (ED), with a mortality ranging from 12 to 56% [1–3]. Shock is defined as inadequate tissue perfusion, decreased cellular perfusion, cellular damage, and metabolic changes, which can result in death. Early recognition and treatment is vital, as mortality remains high even with appropriate treatment [4].

Fluid resuscitation is used as the first line treatment to improve organ perfusion [5, 6], but fluid type, volume and clinical effect can vary considerably [6–8]. The haemodynamic effect of fluid infusion can be short-lived, and studies have found that any increase in blood pressure vanishes within one hour, which may result in liberal use of fluids [9]. Liberal fluid resuscitation, generally more than 5 L over a short period of time, may cause harm, which suggests using a more restrictive approach [10–14]. Restrictive fluid resuscitation, generally less than 2–3 L, seems feasible in both the ED and intensive care unit for patients with sepsis and septic shock [15, 16]. Studies on fluid resuscitation are frequently carried out in patients admitted to the intensive care unit, a subgroup of patients seen with the condition in the ED [17]. However, the CLOVERS trial, primarily investigating liberal versus restrictive fluid resuscitation for hypotensive septic patients in the ED, failed to show benefits of a fluid restrictive approach on patient mortality [18].

Hypotensive patients not responding to fluid resuscitation may require early infusion of vasopressors such as noradrenaline [19, 20]. Data on early initiated noradrenaline therapy has been sparse, but suggest that delaying noradrenaline could increase mortality, which is not yet replicated in larger scale clinical trials [21–24]. The CENSER trial showed that early initiation of noradrenaline increased the proportion of patients achieving shock control within six hours compared to placebo but was underpowered to show any significant decrease in mortality [25].

Investigating noradrenaline therapy, compared to fluid only therapy, could withhold usual standard care in countries which already use noradrenaline as part of their ED management of the affected patient cohorts. As the standard approach is fluid therapy without noradrenaline therapy in Scandinavian EDs, this provides a unique possibility to evaluate early initiation in a noradrenaline naïve setting.

In this protocol, we describe the VASOSHOCK trial, a pragmatic, multi-center, superiority, randomized controlled trial. The trial aims to investigate the impact of initiating early peripheral noradrenaline compared to fluid only therapy in the ED for hypotension and shock, and its implications on shock control and ICU admission.

## Methods and analysis

### Aim

The aim is to investigate whether the use of early initiated noradrenaline therapy compared to fluid therapy alone in non-bleeding hypotensive patients presenting in the ED can improve stabilization of blood pressure for patients, as a surrogate for shock control, and by that reduce the need for ICU admittance.

### Study design and setting

**Fig. 1:**
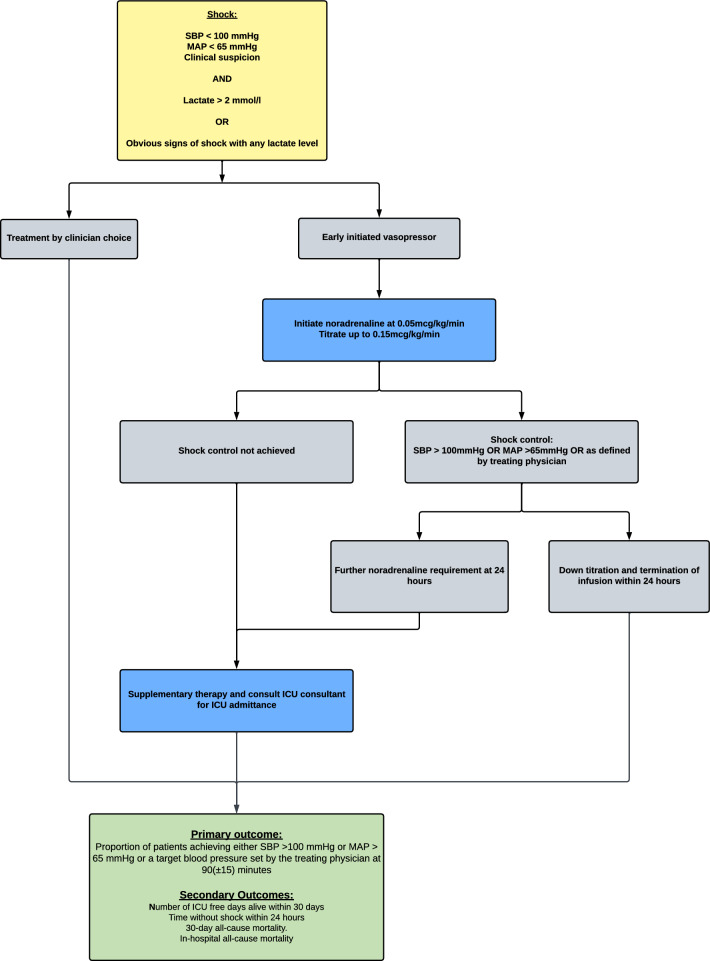
Trial-flow chart. *ICU* intensive care unit, *MAP* mean arterial pressure, *SBP* systolic blood pressure

This study is a pragmatic, multi-center, superiority, randomized controlled trial, randomizing patients 1:1 to either the intervention group, early peripheral noradrenaline in the ED, or the control group, receiving usual care. The trial is planned for recruitment at several hospitals in Denmark and Sweden, with a mix of both university and regional hospitals. Each ED is different in organization and specialty coverage with either a 24/7 presence of an emergency physician or a mix of emergency physicians and medical doctors from other specialties such as internal medicine or general surgery [26–29]. Each ED assesses and treats about 45,000 to 70,000 patients per year. Low acuity patients are commonly handled through outpatient services or out-of-hour services prior to a possible ED evaluation [30]. A trial flow-chart is presented in Fig. 1.

This study protocol is designed in accordance with the Standard Protocol Items: Recommendations for Interventional Trials (SPIRIT) statement and the Consolidated Standards of Reporting Trials (CONSORT) Statement [31, 32].

### Screening of possible participants

Adult patients with either hypotension (defined as systolic blood pressure (SBP) < 100 mmHg or mean arterial pressure (MAP) < 65 mmHg) or with clinical suspicion of shock at any blood pressure level will be assessed for eligibility. Participants will be screened by the site investigators during the patient’s stay in ED. Screened patients will be registered using a web-based survey accessed by a direct link or QR-code through REDCap (Research Electronic Data Capture) provided through the Open Patient data Explorative Network (OPEN) at Odense University Hospital [33]. The trial started screening and recruiting patients in December 2023.

### Inclusion criteria

Participants are included in the trial when fulfilling all the following criteria:

1. At least 18 years of age
2. Signs or suspicion of hypotension or shock (of any type such as septic, vasodilatory or hypovolemic not included in the exclusion criteria) defined as:
  - SBP < 100 mmHg or MAP < 65 mmHg combined with lactate > 2.0 mmol/L,
  - Physician defined blood pressure for the individual patient combined with a lactate > 2.0 mmol/L
  - Either SBP < 100 mmHg or MAP < 65 mmHg with obvious signs of shock with any lactate level evaluated by either two non-specialist physicians (e.g. registrar medical doctors) or one specialist physician.
3. Received at least 500 ml of intravenous fluid before study inclusion (Including prehospital administration)
4. Clinical Frailty Score (CFS) of ≤4. If CFS is ≥5 and the treating physician finds the patient suitable for ICU admittance, the participant can be enrolled, if the on-call ICU doctor would accept the patient for ICU admittance. If the treating physician is unsure of ICU eligibility, regardless of CFS score, the patient should be consulted with the ICU consultant before study inclusion.

### Exclusion criteria

Participants will be excluded if they fulfil any of the following criteria:

1. Cardiogenic, anaphylactic, haemorrhagic, or neurogenic shock suspected by the treating physician.
2. Fertile women (< 60 years of age) with positive urine human gonadotropin (hCG) or plasma-
3. hCG or women breastfeeding
4. Patient deemed terminally ill or with a severe co-morbid status resulting in non-eligibility for ICU admittance decided by either the treating physician or ICU consultant.
5. Severe organ failure outside circulatory failure that requires immediate ICU admission.
6. Known allergy to noradrenaline.
7. Previously enrolled in the trial

### Randomisation and concealment

Patients fulfilling all inclusion criteria, and no exclusions criteria, will be randomized in a 1:1 ratio using block randomization with random block sizes of 2, 4, 6 or 8, stratified by trial site using the randomization model implemented directly in the screening tool through REDCap. Participants are identified using their unique personal Danish or Swedish national identification number and are given a unique trial identification number at the time of inclusion. The randomization allocation sequence is generated by the OPEN data manager and stored securely in relation to the REDCap database hosted at Odense University Hospital, Region of Southern Denmark. Only the data manager or REDCap administrator for the project have access to the allocation sequence if necessary and no other staff participating in the trial will be able to access it.

### Blinding

Full blinding of the intervention is not deemed possible as infusion of noradrenaline is expected to provide a swift and substantial effect on patient blood pressure and other haemodynamic parameters. Participants, investigators, or clinicians will therefore not be blinded to the intervention.

### Study intervention and control treatment

The intervention group will have their usual care supplemented by early initiation of noradrenaline tartrate at a pre-mixed concentration of 1 mg/ml mixed with either isotonic NaCl 0.9% or isotonic glucose 5% with a concentration of 0.06 mg x patient weight in kg to a total volume of 100 ml. Patients with a bodyweight of ≥ 130 kg have their dose calculated as 130 kg to limit substantial concentration increase, which could be incompatible with peripheral infusion.

Infusion is initiated primarily through a peripheral venous catheter (PVC) initiated at a rate of 0.05 microg/kg/min up to a total of 0.15 microg/kg/min for up to 24 h in the ED or subsequent department (such as an intermediate care unit) if such department is participating in the trial. Infusion can be provided through other infusion routes, such as intraosseous, peripherally inserted central catheters or central venous catheters, if this are part of local standard. Infusion rate is titrated by the clinical staff according to a blood pressure aim decided in collaboration by the treating physician and investigator at trial inclusion. Pre-defined targets are either a SBP > 100 mmHg or MAP > 65 mmHg, but other targets can be decided at inclusion if the individual participant might require other target goals than usual, due to known baseline hypo- or hypertension prior to ED arrival. The investigator notes one blood pressure target goal at inclusion in the paper-CRF and the patients electronic patient records. If the decision of treatment goal for the patient is changed at a later point, the initial target goal is still considered the target for outcomes evaluated in the trial.

The blood pressure target is registered in the patient medical records in addition to the paper case report form at inclusion. Patients are monitored with at least non-invasive blood pressure every 15 min combined with continuous 3-lead electrocardiogram (ECG) and peripheral oxygen saturation (SpO_2_). For peripheral lines the staff will also frequently evaluate line placement to identify any possible extravasation events. Patients requiring more than 24-h of noradrenaline therapy, or higher doses than possible in the trial, will be sought admitted to the ICU for further treatment.

The control group will receive usual ED care, where noradrenaline therapy is not available unless initiated by the anaesthesiology and intensive care departments prior to direct transfer to another unit such as the ICU or the operating room. Only necessary data collection, including measurement of blood pressure at 90 min from inclusion, divert from standard treatment.

Patients who otherwise require ICU treatment not possible in the ED, will be transferred to the ICU following usual local guidelines. The trial will not provide any recommendations or restrictions towards concurrent treatment or care decided by the clinical team for either group during the trial and any other treatments are handled by the discretion of the clinical staff. As such, both groups can receive any therapy, including intravenous fluids, if the treating clinicians decides to, without any restrictions or recommendations from the trial. Additionally, patients in the control group can have any treatment initiated during their stay, including noradrenaline therapy in the ICU or other department not otherwise participating in the trial.

There are no restrictions in co-enrolment in other trials and the authors have no current knowledge of on-going or planned studies of the same patient group, which could influence the collected data at any of the participating sites.

### Down-titration and discontinuation of the noradrenaline intervention

Patients in the intervention group will be sought down titrated in the noradrenaline infusion rate during the intervention period if possible. If blood pressure is above the treatment goal, the staff can reduce the infusion rate by 0.01–0.03 microg/kg/min every 15 min and stopped completely if possible. The noradrenaline infusion can be reinstated from inclusion and up to 24 h if new hypotension or shock arises and the patient is still in the participating department. The trial intervention cannot be reinstated if the patient leaves the participating department prior to reaching 24 h post randomization.

### Safety and adverse events

The clinical staff will, in collaboration with the investigators, assess adverse and serious adverse events and reactions in relation to the trial following the EU regulation [34]. Serious adverse events are either resulting in the death of the participant, a life-threatening situation, requires hospitalization, prolongation of the hospitalization or results in either significant disability or incapacity of the patient.

In previous trials investigating fluid therapy and vasopressors such as noradrenaline for shock, serious adverse events and reactions were significantly lower than in the standard care groups. CENSER had significantly more events in the standard care arm experiencing cardiogenic pulmonary oedema, 27.7% vs. 14.4%, RR 0.70 (0.56–0.87) and new onset cardiac arrhythmia, 20% vs. 11%, RR 0.74 (0.56–0.94) with no difference for events such as skin necrosis, limb or intestinal ischemia [25]. CLOVERS found a comparable 90-day mortality, with no significant difference between groups for ventricular arrhythmias [18]. An earlier study, the SEPSISPAM trial investigating lower versus higher blood pressure targets for patients in septic shock had patients experiencing atrial fibrillation, ventricular fibrillation or tachycardia, digital and mesenteric ischemia among other serious adverse events [35]. Only atrial fibrillation was higher in the group with higher target pressures and as the trial was investigated in ICU patients, with a high proportion of mechanical ventilated patients with far higher doses than under investigation in this trial, the risks might not be comparable.

The trial is therefore conducted through a risk-based approach evaluating extravasation and overdose events during the treatment period in the intervention group, while also investigating acute kidney injury and pulmonary oedema for both groups. Additionally, any suspected unexpected serious adverse reactions are registered as necessary and required by the authorities. Clinical deterioration in participants due to their critical illness but not related to the trial interventions are for registration purposes, are not considered a serious adverse event. Other adverse or serious adverse events and reactions are not systematically registered in the trial, but investigators can at any time report any event if they deem it necessary.

#### Extravasation

Extravasation is handled using the following approach: Discontinue the infusion and restart in another non-extravasated line (such as a PVC) at the same infusion rate. Retract medication in the dislodged, or extravasated, catheter until blood is drawn. Inject phentolamine 1 mg/ml up to a total of 5-10 ml partly through the extravasated catheter and/or at the extravasation site including the borders of affected area. Elevate the affected extremity as long as deemed necessary and possible in consideration of the patient’s ability and clinical status. Apply warm dry dressings 3–4 times a day for the first 24–48 h. Any subsequent suspicion or signs of tissue necrosis should be evaluated by the investigators, and if necessary, in collaboration with the surgical departments, preferably plastic surgery.

#### Overdose

Overdose is handled with immediate discontinuation of the infusion, and retraction of the medication in the dislodged or extravasated catheter until blood is drawn. The catheter is removed, and the patient is observed for at least 15 min, receiving supplemental care as needed, before anti-hypertensive treatment to ensure full clearance of the noradrenaline effect. The clinical staff are recommended to investigate other possible causes of the adverse reaction if the reaction persists beyond the 15-min mark.

### Staff education

All investigators participating in the trial will be required to follow education in the trial, including the use of noradrenaline therapy in the setting of shock, with special emphasis on handling noradrenaline, including peripheral infusion. This includes investigation and treatment of any adverse events and reactions as previously described. Additionally, any nursing staff who participate in the care and treatment of patients included in the trial also receive necessary education on handling of the medication following the trial standard operating procedures. This includes necessary knowledge to suspect possible adverse events and reactions, who they are obligated to contact the investigators about, if such suspicion arises.

### Outcomes

The primary outcome is proportion of patients achieving either SBP > 100 mmHg or MAP > 65 mmHg or a target blood pressure set by the treating physician at 90(± 15) minutes after inclusion.

Key secondary outcomes include number of ICU free days alive within 30 days, time without shock within 24 h, 30-day all-cause mortality and in-hospital all-cause mortality.

Exploratory outcomes include.

- Proportion of patients receiving vasopressor at any point within 24 h.
- Time to vasopressor initiation during hospitalization
- Duration of vasopressor infusion during hospitalization
- Re-admission for any reason within 30 days of inclusion
- ED length of stay
- Proportion of patients admitted to the ICU during hospitalization
- Hospital length of stay
- ICU length of stay during hospitalization
- Need for mechanical ventilation (either invasive or non-invasive ventilation) within 30-days
- Need for renal replacement therapy (continuous renal replacement therapy or dialysis) within 30-days
- Organ support-free days within 30 days (defined as mechanical ventilation, vasopressor or inotropic therapy, or dialysis).
- Amount of fluid therapy received within the first 24 h

Safety outcomes include.

- Proportion of patients developing pulmonary oedema at any point within 72 h from randomization (Diagnosed by physician in accordance with local guidelines, e.g., clinical decision including evaluation with paraclinical imaging such as x-ray or lung ultrasound)
- Proportion of patients developing acute kidney injury at any point within 72 h from randomization (Defined as an absolute increase of creatinine ≥ 26.5 µmol/L or ≥ 1.5 fold from baseline)
- Proportion of patients experiencing extravasation of peripheral noradrenaline
- Proportion of patients having serious complications due to extravasation (Defined as a serious complication fulfilling the criteria for a serious adverse reaction, e.g. skin necrosis necessitating surgical intervention)
- Proportion of patients experiencing overdosing due to noradrenaline infusion in the trial (Defined as severe hypertension, and reflex bradycardia suspected by the staff or investigators)
- Proportion of patients experiencing any SAE, SAR or SUSAR related to the trial intervention or procedures registered during the trial

### Sample size calculation

The sample size is based on data from the CENSER trial [25]. The median time from “initial treatment to achieving the target of mean arterial blood pressure + tissue perfusion goal” for the intervention group was 4 h:45 min and 6 h:02 min for the controls. Given this difference, and considering a log normal distribution of the data, an alpha value of 0.05, and power of 90%, a sample size of 80 persons per groups is necessary [36]. As our target is evaluated earlier, with an included higher blood pressure target while allowing the treating physicians to individualize treatment goals for the patients, and to further investigate the key secondary outcomes, the planned number of participants is doubled to 320 participants with 160 participants in each group.

### Data collection and protection

Data collection will be completed and managed using a secure web-based software platform REDCap. All data will be entered using the secure web-form and from there extracted for subsequent data analysis. The clinical staff will use a paper Case Report Form (paper-CRF) to register bed-side data during the trial intervention period for both groups, which includes registrations of parameters such as noradrenaline infusion rate, blood pressure measurements, fluid therapy type and volume and other trial related data. After completion of the intervention period, the paper-CRF data is entered into and then uploaded to the electronic CRF (eCRF). Subsequent data is registered by the research staff directly in the eCRF. All access to data will be logged on a person level complying with the European code for handling person data and according to national law [37–39].

### Statistical analysis plan

**Fig. 2:**
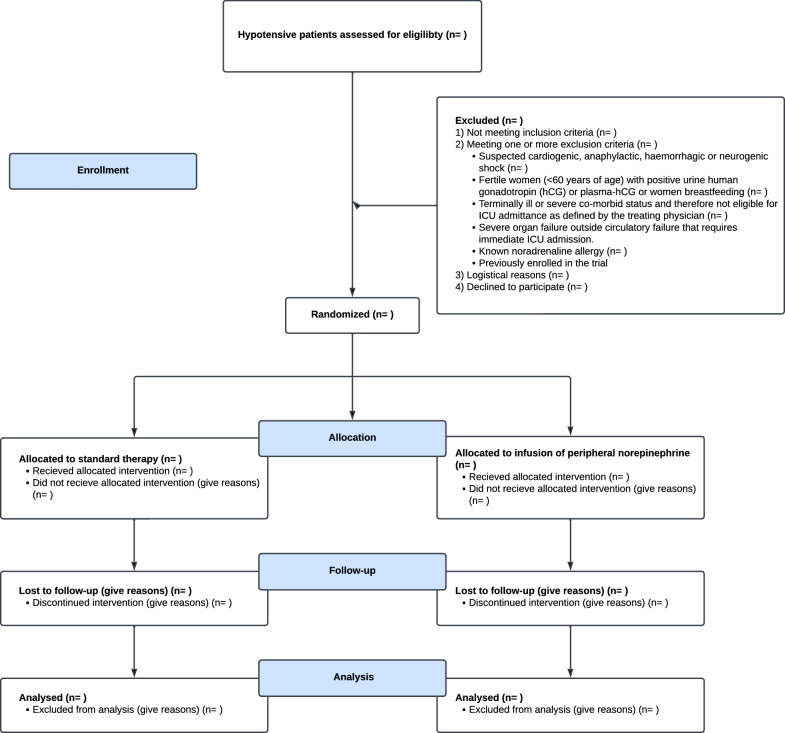
CONSORT diagram draft. *ICU* intensive care unit

The primary outcome, and dichotomous secondary and tertiary outcomes, will be reported as proportions with 95% confidence intervals (CIs) for both arms, and compared by estimating a relative risk (RR) with 95% CIs and p-value by logistic regression followed by prediction of absolute risk via G-computation. Continuous outcomes will be reported as means and mean difference between groups with 95%CI and compared by linear regression. To consider the expected non-normality of data, 95%CIs and p-values will be determined by non-parametric bootstrapping. The primary outcome and mortality will also be presented using Cox regression and Kaplan–Meier curves. Analyses will be adjusted for pre-specified covariates of site allocation, age, lactate level at inclusion and trial site. The main analysis will be intention-to-treat with per protocol as sensitivity analysis. Additionally, a Bayesian sensitivity analysis for both primary and secondary analyses will be conducted. Missing data will be presented descriptively in the final analysis and is not expected to be present in the primary or secondary outcomes. There is no pre-planned weighting or imputation of missing data. Patient in- and exclusion will be presented as a CONSORT diagram (Fig. 2) [32]. Statistical analyses will be performed using R 4.4.2 (R Core Team 2024, Vienna) or newer version if such is published before the conducted analyses is performed. The statistical analysis plan will be finalized prior to inclusion of the last participant.

### Early termination of the trial

The trial can be terminated early in the following circumstances:

1. The Sponsor receives a substantial number of serious adverse events or reactions leading to a concerning level of risk for the patients,
2. The trial is no longer feasible at any of the trial sites due to local organisational or clinical implications, such as unavailability of noradrenaline,
3. Slow inclusion rate at trial sites leading to a problematic timeframe to complete the study, such as double required time to complete the study,

### Data monitoring and interim analyses

Due to the design of the pragmatic approach and size of the trial, there are no formalised data monitoring committee nor any prior planned interim analysis of the trial.

### Clinical trial monitoring and good clinical practice

Danish trial sites will be monitored by the regional Good Clinical Practice monitoring unit affiliated with OPEN at Odense University Hospital. Swedish study sites will be monitored by Clinical Studies Sweden. The monitors are not otherwise affiliated with the trial.

### Patient and public involvement

Patients have been interviewed in two emergency departments using an unstructured interview approach on their considerations for implementation of the trial in relation to the described research aims before the first protocol was finalised. A patient- and relative representative has been involved since the planning phase of the trial and assists with ongoing perspectives relevant to the participants of the trial. This includes insight and suggestions towards any parts of the trial, though outcomes, safety, and ethical considerations. The patient- and relative representative has been part of the revision of the informed consent forms for both patients and their relatives prior to ethical committee approval of these forms.

## Discussion

Early establishment of necessary treatment of patients with shock is essential to reduce mortality and morbidity [40]. The timing of these treatments is not thoroughly investigated though substantial amounts of research is conducted each year [41–43]. Research in shock is often related to specific conditions such as septic, cardiogenic, or haemorrhagic shock, but treatment algorithms for patients with undifferentiated shock is often extrapolated from these conditions [15, 40, 44–47]. As such, the evidence for the treatment of septic shock, often following the Surviving Sepsis Campaign recommendations, is frequently used for the treatment of undifferentiated shock patient where haemorrhage, cardiogenic or anaphylactic cause is not suspected initially [47–49]. The research on fluid therapy in resuscitation of shock is extensive, and more restrictive approaches are now commonly recommended [19, 50, 51]. Timing of vasopressor treatments are still lacking, though several studies, primarily observational, suggests that an earlier intervention are associated with better outcomes [52–55]. The use of peripheral administration of vasopressor therapy has also been evaluated in different clinical settings showing high safety if handled correctly [56–61], while also providing shorter time to vasopressor initiation [19, 62, 63]. Research on the use of noradrenaline in settings where it is not routinely used is limited, as current trials typically report on ICU settings where vasopressor or inotropic treatments are standard practice. The few studies conducted in EDs often come from international settings where vasopressors are commonly used during the resuscitation of critically ill patients, in contrast to Scandinavian EDs, where such treatments are not implemented [18, 25, 62, 64]. This provides a situation where a pragmatic trial design is reasonable [65, 66]. As the use of noradrenaline therapy is not previously established as a commonly used treatment in Scandinavian ED’s, it provides a unique opportunity to not only investigate the use of early initiation of these treatments but also provide insight with a setting where noradrenaline therapy has not previously been provided.

### Type of shock and criteria in the trial

The trial includes patients with suspected or obvious signs of shock as described in the inclusion and exclusion criteria. The trial does not specify criteria for suspected or obvious signs of shock other than the mentioned use of blood pressure and lactate in the trial. As the diagnosis of shock can be difficult and does not rely on one single parameter [4, 67–69]. The decision on whether the patient fulfils these criteria of suspicion of or obvious signs of shock, relies on the staff assessing the patient for possible inclusion. This would include a combination of clinical and paraclinical investigations, including aspects of blood pressure, clinical presentation, blood tests including acid–base disturbances, point-of-care ultrasound among others. This can hamper the reproducibility of the trial, but we believe this is more in line of the reality of treating patients with shock in the early stages during ED admission.

As patients included in the trial arrives and are treated in the ED, the knowledge of causes and possible categorization of the shock condition can difficult, if not impossible, at an early stage. Therefore, the trial will include patients with several different possible causes of shock. If the clinical staff and investigators suspect a possible cause listed in the exclusion criteria (e.g. anaphylactic) the patients are excluded during the screening process. However, if patients are included in the trial during the early stages of their shock where specific evidence of the cause is not present, but any of the exclusion causes are later identified during the hospitalisation, they will still participate in the trial and planned follow-up. In comparison, previous studies has suggest that the correct identification of sepsis for patients admitted to the ICU is often incorrect [70], and multifactorial shock in different patient cohorts are not uncommon, both in septic, cardiogenic or trauma related scenarios [71–73]. The use of noradrenaline in the state of hypovolemic is not unusual, but still sparsely investigated [74–76]. However, as the trial does not restrict any fluid therapy, patients which still require fluid therapy for their condition can receive these as decided by the clinical team.

The decision to not include a lactate cut-off for all patients at inclusion, was decided as patients in shock might present with normolactatemia while critically ill, even though elevated lactate is a significant predictor for illness severity and mortality risk [77, 78]. Definitions of shock varies broadly between causes of the condition, where certain types such as shocked due to adrenal crisis, vasoplegia, cardiogenic shock and anaphylaxis does not strictly dictate hyperlactatemia as part of the criteria [79–83]. This contrasts with the SEPSIS-3 definition of septic shock, where hyperlactatemia is now a requirement for the condition [84]. This was not the case in the SEPSIS-2 definition, where there was evidence of other organ failures [85]. As the specific cause of shock might not be identified at the time of inclusion, the clinicians can include patients that show obvious signs of shock regardless of lactate levels.

### Limitations

This trial is not without limitations. As the intervention is not placebo blinded, there is a high risk of implementing bias into the trial due and by that, possibly damaging the interpretation of the findings. The choice of not blinding the trial relies on the expected effects of noradrenaline infusion. Most patients are expected to have a rather swift response to noradrenaline infusion, for some even at lower infusion rates, essentially unblinding the treatment as compared to a similar infusion of a placebo treatment. Current recommendations for pragmatic trials also suggest not blinding treatment arms unless specific circumstances necessitate this approach [65, 66].

The possible use of non-invasive blood pressure measurements is not unproblematic in the setting of shock, as its use could lead to overestimation of blood pressure and by that, providing insufficient treatment for the patients. However, the use of invasive measurement methods is not currently feasible in most Scandinavian EDs, and previous studies have suggested that non-invasive blood pressure is sufficient [86].

#### Choice of primary outcome

The primary outcome is a clinician targeted blood pressure, as a surrogate for shock control, rather than patient targeted outcome such as mortality. The choice for this is due to substantial lack of current national data on the number of patients with shock being ICU candidates, and how long these patients’ length of stay in the ICU are. Due to this, it was not feasible to calculate a sample size that considered ICU length of stay and survival as a combined endpoint, with a reasonable certainty of the necessary participant enrolment. The possibility to provide a sample size appropriate for an investigation of mortality is not feasible in the setting of this trial, as sample sizes which could effectively evaluate mortality changes in the population, are not not achievable in our current setting.

The authors acknowledge that a sole blood pressure target does not constitute a full definition for stabilization of shock, which would also rely on other parameters, such as capillary refill time, urine production or lowered lactate levels. The CENSER trial had a primary outcome of a MAP ≥ 65 mmHg with evidence of adequate tissue perfusion Adequate tissue perfusion were defined as a urine flow of more than 0.5 ml/kg/h for 2 consecutive hours, or decreased serum lactate of > 10% compared to the initial lactate level. CENSER did find a statistically significant higher proportion of patients in the intervention group compared to the control group. However, this was only seen in outcomes where urine output by 6 h were a part of the evaluation, not when MAP and lactate was the only part of the evaluation. Patients only achieving the target MAP was significantly higher in the intervention group of 85.6% vs. 67.1% in the control group at 6 h [25]. This might not be the case in this trial, as the ED cannot provide open-label vasopressor therapy and patients would therefore need to be admitted to the ICU for this.

As the VASOSHOCK trial is conducted as a highly pragmatic trial, the primary was chosen so it was possible to provide an educated calculation of a possible stabilization indication, which would not require 24-h presence by research staff for data collection. Also, as patients are also included where the clinicians finds obvious signs of shock, with normal lactate levels, the use of lactate normalization is not possible as part of the primary outcome assessment. The primary outcome was therefore decided to be a target blood pressure target decided at the time of inclusion as a surrogate for shock control, rather than a combination blood pressure and tissue perfusion parameters. We therefore reached a sample size of 160 patients (80 in each group) as the primary outcome goal as described in this protocol and doubled this to 320 patients to possibly provide enough statistical power for some of the secondary or tertiary outcomes.

As of note, the measured blood pressure at 90(± 15) minutes is usually from non-invasive measurements, and therefore not with several same minute measurements as usually possible with arterial cannulation. If several measurements are registered, the blood pressure closest to 90 min from inclusion is used as the evaluation of the outcome.

## Conclusion

In summary, the VASOSHOCK trial is a pragmatic, multi-center, superiority, randomized controlled trial investigating early initiation of peripheral noradrenaline in hypotension and shock, investigating the impact of this treatment on blood pressure stabilisation as a surrogate for shock control, reducing ICU admittance, length of stay and possibly, mortality.

## Supplementary Information

Supplementary material 1.

## Data Availability

The datasets generated and/or analyzed during the current study are not publicly available due to Danish regulations [37, 87]. Trial protocol, statistical analysis plan, standard operating procedures and other related trial documents is available through the corresponding author upon reasonable request.

## Abbreviations

AE: Adverse event
CFS: Clinical frailty scale
ECG: Electrocardiogram
ED: Emergency department
ICU: Intensive care unit
MAP: Mean arterial pressure
OPEN: Open patient data explorative network
OUH: Odense university hospital
PVC: Peripheral venous catheter
REDCap: Research electronic data capture
SAE: Serious adverse event
SAR: Serious adverse reaction
SBP: Systolic blood pressure
SD: Standard deviation
SpO_2_: Peripheral oxygen saturation
SUSAR: Suspected unexpected serious adverse reaction
